# Does the key to treat Rheumatoid nodules lie with oncology? - Is Cisplatin an option?

**DOI:** 10.1186/1471-2474-14-S1-A5

**Published:** 2013-02-14

**Authors:** Eisha K Omar, Suresh Adimulam

**Affiliations:** 1 Wansbeck General Hospital, Ashington, UK

## Case

Rheumatoid nodules (RN) are not only unsightly and functionally limiting but also indicate poor prognosis in rheumatoid arthritis (RA) [1]. Treatment options are limited as traditional disease modifying antirheumatic drugs (DMARDs) like Methotrexate [2], Cyclosporine [3], Leflunomide [4] and anti TNF therapy [5,6] can make RN worse. RN can also recur after intranodular steroid injections [7] or surgical excision [8].

We report the case of a forty three year old man who was diagnosed with RA at the age of 26 years however was started on sequential DMARDS only four years later upon referral to Rheumatology, by which time RN had developed on his elbows. He was found to have seropositive, erosive RA and was started on Sulfasalazine, and a few months later oral Methotrexate and Hydroxychloroquine were added sequentially. Despite these measures, his arthritis was sub-optimally controlled and his RN continued to deteriorate.

In November 2009 he was diagnosed with head and neck squamous cell carcinoma involving his neck, nasal cavity and tongue. All DMARDs were discontinued on diagnosis. He received a six week course of Cisplatin as a single chemotherapeutic agent in Jan/Feb ’10 in addition to local radiotherapy.

He noticed that his RN started to disappear during chemotherapy. His arthritis has been in complete remission after his course of cisplatin and has not noticed any recurrence of RN despite remaining off all DMARDs till November 2011.

## Discussion

Our patient had sustained remission of RA and complete disappearance of RN with Cisplatin which has not been achieved with other treatment modalities e.g, Tacrolimus [9] and Colchicine [10]. Although D-penicillamine has shown to be effective in treatment of RN [11,12] it is not very effective in controlling RA, hence the need for further studies of Cisplatin in the management of RA and RN.
